# Shrinking the malaria map in China: measuring the progress of the National Malaria Elimination Programme

**DOI:** 10.1186/s40249-016-0146-5

**Published:** 2016-05-19

**Authors:** Tao Hu, Yao-Bao Liu, Shao-Sen Zhang, Zhi-Gui Xia, Shui-Sen Zhou, Jun Yan, Jun Cao, Zhan-Chun Feng

**Affiliations:** School of Medicine and Health Management, Tongji Medical College, Huazhong University of Science and Technology, Wuhan, Hubei People’s Republic of China; Bureau of Disease Prevention and Control, National Health and Family Planning Commission of the People’s Republic of China, Beijing, People’s Republic of China; Jiangsu Institute of Parasitic Diseases, Wuxi, Jiangsu People’s Republic of China; National Institute of Parasitic Diseases, Chinese Center for Disease Control and Prevention, Shanghai, People’s Republic of China; Public Health Research Center, Jiangnan University, Wuxi, People’s Republic of China

**Keywords:** Malaria elimination, Incidence, Progress, Experiences, Challenges, China

## Abstract

**Background:**

Remarkable progress has been made towards the elimination of malaria in China since the National Malaria Elimination Programme (NMEP) was launched in 2010. The incidence of locally-acquired malaria cases has declined rapidly and endemic areas have also dramatically shrunk. In total, 3 078 malaria cases were reported in 2014, but only 56 cases were indigenous. In order to further promote the elimination programme, we reviewed the progress of and experiences associated with malaria elimination in China, and identified the challenges and priorities for the next stage of the programme.

**Methods:**

Data were collected from the web-based China Information System for Disease Control and Prevention, and the China Annual Report of Malaria Elimination. The progress towards the elimination of malaria from 2010 to 2014 was measured.

**Results:**

During the implementation of the NMEP from 2010 to 2014, local malaria incidence has declined continuously, only remaining in the Yunnan Province and Tibet Autonomous Region in 2014. By the end of 2015, 75.6 % (1 636/2 163) of the malaria-endemic counties passed the sub-national elimination assessment. The main challenges are cases of border malaria and imported malaria from other countries. Sustainable support and investment from the government, the establishment of an effective surveillance and response system, and risk assessments for the potential reintroduction of malaria are priorities for the next stage of the elimination programme.

**Conclusions:**

The NMEP in China has been successfully implemented thus far and the malaria map has shrunk dramatically. The priorities for malaria elimination are interventions to block transmission at border areas, management of imported malaria cases, preventing malaria reintroduction, capacity building, and sustainability of malaria surveillance and response.

**Electronic supplementary material:**

The online version of this article (doi:10.1186/s40249-016-0146-5) contains supplementary material, which is available to authorized users.

## Multilingual abstracts

Please see Additional file 1 for translations of the abstract into the six official working languages of the United Nations.

## Background

Malaria remains one of the most serious public health issues in the world. According to the latest available data, about 3.2 billion people were at risk of contracting malaria in 2015, and an estimated 214 million new cases and 438 000 deaths associated with malaria were reported that same year [1]. Historically, malaria was extensively endemic in China and large-scale outbreaks occurred in the 1960s and 1970s, which had a serious impact on people’s health and inhibited socio-economic development [2, 3]. After intensive efforts for many years, malaria has been effectively controlled in China through the implementation of effective strategies and measures, such as mass malaria control activities, integrated vector control and joint control strategies. The intensity of malaria epidemics and the number of endemic areas have decreased significantly, and falciparum malaria transmission was successfully interrupted in central China in early 1990s [2, 4, 5]. In 2010, the number of malaria cases nationwide was 7 855, a reduction of 45.8 % compared with the 14 491 cases reported in 2009. Over 95 % of the counties in China have a malaria incidence below 1/10 000 [6].

In 2008, the United Nations issued the Millennium Development Goals, one of which was to globally eradicate malaria. Many countries have made remarkable progress in malaria elimination and 109 countries were malaria free by 2010 [7]. To respond to this global action plan, the Chinese government developed a National Malaria Elimination Programme (NMEP) in 2010, and the Action Plan of China Malaria Elimination (2010–2020) (APCME) was officially endorsed in the same year by the Ministry of Health in conjunction with 12 other ministries including Finance, Education, Science and Technology, Entry-Exit Inspection and Quarantine, and so on. The general goal of the NMEP is to “eliminate local malaria transmission except in some of the Yunnan-Myanmar border areas by 2015 and realize malaria elimination across China by 2020” [8].

Since the launch of the elimination programme in 2010, along with socio-economic development, progress of urbanisation, and changes in the natural environment and malaria vectors, the incidence of locally-acquired malaria in China has declined sharply and malaria-endemic areas have also dramatically shrunk. There were 3 078 malaria cases reported in 2014, but only 56 were indigenous cases [9]. China is currently on the path towards malaria elimination and has developed a roadmap for nationwide elimination of the disease [10]. In order to understand the current status of the NMEP and ensure the realisation of the final goal of malaria elimination countrywide in 2020, the achievements and experiences of the past five years since the launch of the programme are reviewed in this paper. Challenges and priorities for the next steps are also identified.

## Methods

### Brief profile of China

China is located in East Asia and the Western Pacific Region. Covering approximately 9.6 million square kilometres, China has a population of 1.37 billion, with an average population density of 145 per square kilometre in 2014. China is divided into provinces, autonomous regions and municipalities (P/A/M) directly under the administration of the Central Government. Currently, the country is divided into 23 provinces, five autonomous regions, four municipalities and two special administrative regions [11].

### Data collection

Data on the NMEP from 2010 to 2014 were collected and the progress towards the elimination of malaria in China was reviewed. Data were obtained from the web-based China Information System for Disease Control and Prevention (CISDCP) and the China Annual Report of Malaria Elimination. Data collected included the number of reported malaria cases sorted by indigenous cases and imported cases from other countries; strategies and interventions implemented, including number of blood examinations conducted, distribution of long-lasting insecticide-treated nets (LLINs) and indoor residual spraying (IRS), provision of training, administering of health education; and amount of financial investment. Data from Hong Kong, Macao and Taiwan were not included in this analysis.

### Data analysis and mapping

Data were double entered into Microsoft Excel 2007 (Microsoft Corporation, Redmond, WA, USA) and then a descriptive analysis was conducted. The maps showing the geographical distribution of indigenous malaria cases were generated using ArcGIS software version 10.1 (Environmental Systems Research Institute. Inc., Redlands, CA, USA).

## Results

### Implementation of malaria elimination strategies and interventions

Since the launch of the NMEP, the Chinese government has been massively promoting the implementation of malaria elimination strategies and interventions (see Table 1). A special fund from central government finance was set up for malaria and the total amount given from 2011 to 2014 was 426 million CNY (US$ 66.6 million).

**Table 1:** Implementation of strategies and interventions for malaria elimination in China, 2010–2014

Year	Blood examination (Persons)	LLINs (Nets)	IRS (Persons covered)
2010	7 115 784	1 030 373	854 701 275
2011	9 189 270	1 840 792	1 043 963
2012	6 918 657	251 555	1 092 158
2013	5 554 960	58 874	447 639
2014	4 403 633	19 899	504 936
Total	33 182 304	3 201 493	857 789 971

A total of 33 182 304 blood tests were performed using both passive and active case detection for fever patients, with 22 277 malaria cases found from 2010 to 2014. By the end of 2014, the proportion of suspected malaria cases that received parasitological tests and the proportion of reported malaria cases based on parasitological confirmation reached 99.68 and 98.15 %, respectively. The proportion of cases reported within one day (24 h) after diagnosis, cases investigated within three days after reported, and foci investigation and action conducted within seven days after case reported reached 100, 98.95 and 100 %, respectively in 2014.

A total of 3 201 493 LLINs were distributed and 857 789 971 people were protected by IRS during the period from 2010 to 2014. Trainings that mainly targeted public health staff, clinicians and microscopists were carried out at different levels. About 130 000 technicians were trained in malaria microscopy during the period from 2010 to 2014. Health education campaigns for residents and students were carried out combined with the National Malaria Day (April 26th) every year across China. At present, an effective surveillance system that combines routine surveillance and sentinel surveillance has been established. The routine surveillance includes a case reporting system based on the web-based CISDCP and a web-based information system specifically designed for parasitic diseases. A total of 49 sentinel sites have been set up, which covered 25 P/A/M across China, to provide information and technical support for malaria elimination in China. The activities of the sentinel sites include active case detection, vector surveillance (species, population density and insecticide resistance) and anti-malarial drug efficacy. A laboratory network for malaria diagnosis has been developed [12] and 22 provincial-level malaria diagnosis reference laboratories in 24 malaria-endemic provinces were established by the end of 2014.

### Indigenous malaria incidence has continuously been declining

**Fig. 1:**
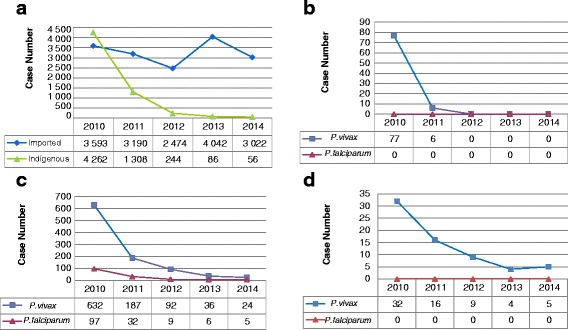
The changing malaria situation in China, 2010–2014. **a** Number of malaria cases (indigenous versus imported) reported in China. **b** Number of indigenous malaria cases (*P. vivax* versus *P. falciparum*) reported in Hainan Province. **c** Number of indigenous malaria cases (*P. vivax* versus *P. falciparum*) reported in Yunnan Province. **d** Number of indigenous malaria cases (*P. vivax* versus *P. falciparum*) reported in Tibet

Since the launch of the NMEP, indigenous malaria incidence has declined significantly. A total of 4 262 indigenous cases were reported in China in 2010, however, only 56 cases were reported in 2014, a reduction of 98.6 % (see Fig. 1a). There was only one county (Motuo County in the Tibet Autonomous Region) that had an incidence of indigenous malaria of more than 1/10 000 in 2014. Hainan Province was the most seriously affected malaria-endemic area, as it had the highest transmission of *Plasmodium falciparum* and *P. vivax* malaria in history [13], however, no locally-acquired falciparum malaria cases have been reported since 2010 and the number of vivax malaria cases has declined sharply to zero since 2012 (see Fig. 1b). The number of indigenous malaria cases in Yunnan Province has continuously been decreasing with only five falciparum cases and 24 vivax cases reported in 2014 (see Fig. 1c). The number of indigenous malaria cases in the Tibet region also declined steadily from 2010 to 2014 (see Fig. 1d).

### Malaria-endemic areas have shrunk dramatically

**Fig. 2:**
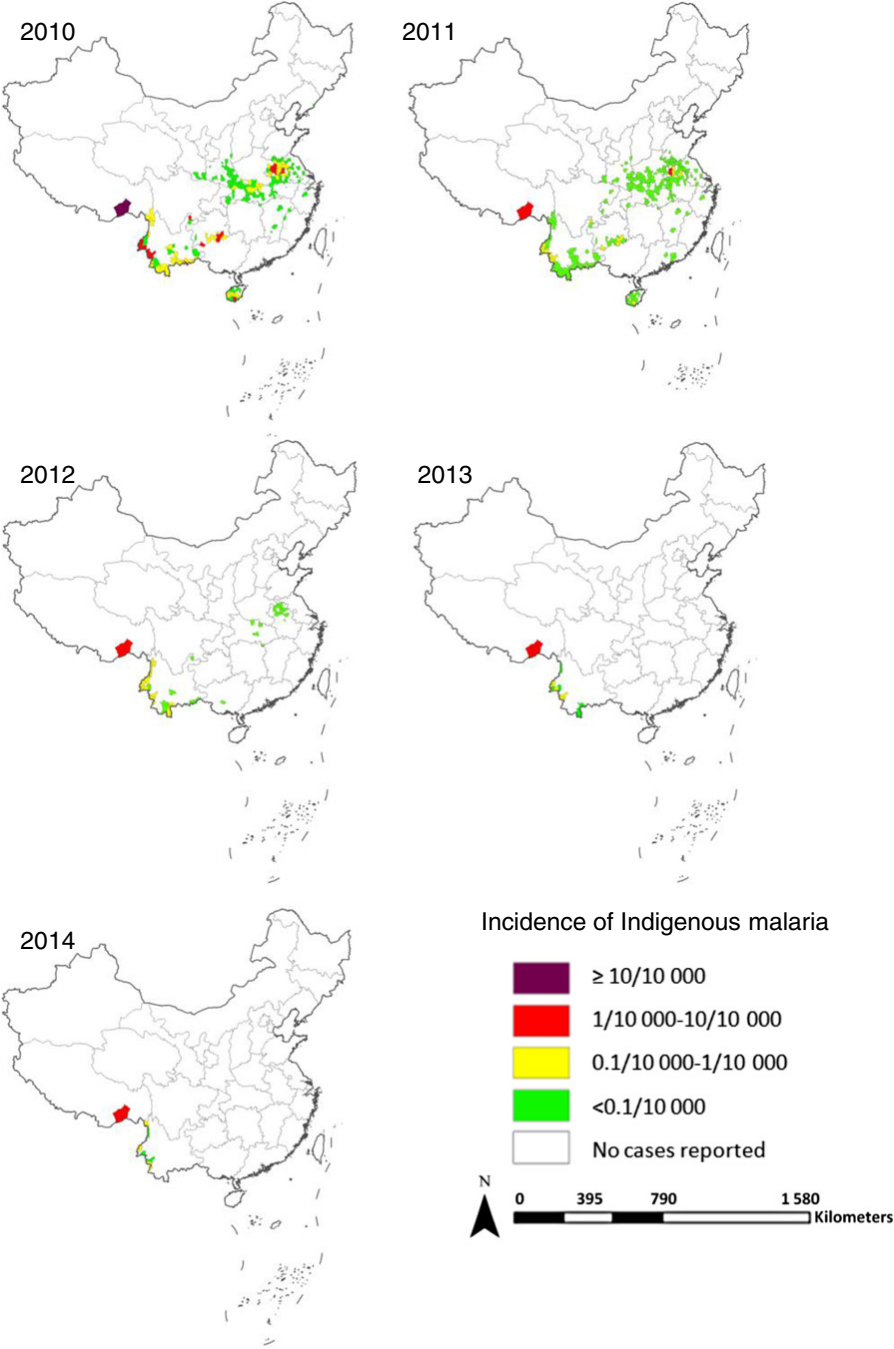
Changes in the distribution of indigenous malaria cases in China between 2010 and 2014

In total, 2 194 counties in 24 provinces across China were identified as malaria-endemic counties and 762 counties reported local malaria cases when the elimination programme was first launched in 2010. The number of counties with local cases decreased to 155, 41, 12 and 10 by the end of 2011, 2012, 2013 and 2014, respectively. Only Yunnan Province and Tibet Autonomous Region reported locally-acquired malaria cases by the end of 2014, with the cases mainly distributed in nine counties along the China-Myanmar border and one county (Motuo County) in the Tibet Autonomous Region (see Fig. 2).

### Progress towards malaria elimination

**Fig. 3:**
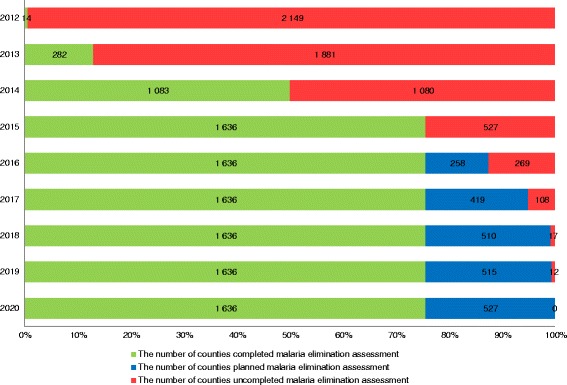
Progress of the sub-national malaria elimination assessment in China

To ensure progress towards malaria elimination, a sub-national elimination assessment was conducted starting from 2012. The assessment of each county was carried out by up usually prefecture-level authority, according to the Criteria for Control and Elimination of Malaria (GB26345-2010) and the protocols developed by the National Health and Family Planning Commission of China. By the end of 2015, 75.6 % (1 636/2 163) of malaria-endemic counties passed the assessment and were officially recognised as reaching the goal of malaria elimination. All other counties are expected to be assessed by the end of 2020, as according to the APCME (see Fig. 3).

## Discussion

The Global Technical Strategy has proposed to eliminate malaria in 35 new countries by 2030 [14]. At the Ninth East Asia Summit, regional leaders agreed to the goal of an Asia Pacific free of malaria by 2030 and proposed a roadmap for malaria elimination [15]. Malaria elimination in China is one of the most important goals of global malaria elimination and is of great concern to the international community [16, 17]. Recent progress made by the NMEP has shown that remarkable strides can be made with adequate investment and the proper strategies.

Overall, the NMEP in China is proceeding as planned and the malaria map has successfully been shrunk to limited border areas. This success is driven by several factors. Firstly, it has benefited from the attention and strong political commitment of the Chinese government. The APCME proposed clear objectives, strategies, measures and schedules for malaria elimination at the national level from the beginning of the programme, and the Chinese government has continued to financially support malaria elimination strategies despite the Global Fund to Fight AIDS, Tuberculosis and Malaria stopping its funding for China in 2012 [18]. Secondly, regional collaboration, which is called China’s joint malaria prevention and control mechanism, has largely helped the progress towards malaria elimination. The joint mechanism, which is under the guidance of the different levels of governments and health authorities, groups together regions adjacent to each other with similar natural and geographical conditions, consistent transmission intensities and epidemic factors. These regions then implement the same strategies through unified planning and conduct simultaneous actions, joint training, unified monitoring and evaluation, as well as hold regular meetings to exchange experiences [19]. In addition, cooperation between China and neighbouring countries including Myanmar, Laos and Vietnam has played an important role in improving control of malaria at cross-border areas and solving the cross-border challenge of imported malaria. Thirdly, the national web-based case reporting system combined with a specific system for parasitic diseases provides a strong support for data collection. Fourthly, the strategy for surveillance and response called “1-3-7” has been developed and implemented in China since early 2012, and has played an important role in efficiently detecting, treating and responding to individual malaria cases and eliminating the source of infection promptly [20–23]. In addition, a lot of training has been conducted to strengthen capacity building. For example, a national competition measuring the skills of microscopists has been held every year since 2011, which has improved the competency of microscopists at different levels and provided strong technical support for malaria elimination [24, 25].

Challenges to achieve the final goal of a malaria free China by 2020 are mainly related to cross-border and imported malaria. There are 25 counties along the 4060-km borderline of Yunnan Province with Myanmar, Laos and Vietnam. The border areas belong to a mixed endemic area with transmission of *P. falciparum* and *P. vivax* malaria throughout the year. The natural environment in these areas is complex and a variety of malaria vectors, such as *Anopheles dirus* and *A. minimus*, usually co-exist in one setting and have a high vector capacity for transmitting malaria [26–28]. Furthermore, there is a large cross-border mobile population, as there is no natural barrier in China-Myanmar border areas and this makes management of imported malaria a great challenge [29, 30]. The other border area is Motuo County in Tibet Autonomous Region, which is one of the poorest areas in China and borders with India. The population of Motuo is only 10 000, but the county’s ability to carry out a malaria elimination programme is limited due to the poor quality transportation and health system. Furthermore, because basic information including malaria epidemiology, biology of local parasites and vectors still remains poorly understood in Motuo, there are currently no effective strategies and measures for malaria elimination in this county [9, 31].

Another challenge is the increase of imported malaria cases from other countries, especially from Africa. Local malaria has been effectively controlled in most areas of China, however, with the increase of China’s cross-border trade and foreign aid projects, imported cases have increased rapidly in recent years [9, 32, 33]. An outbreak with more than 874 imported cases even occurred in Shanglin County from May to August 2013 [34]. Almost all of the P/A/M (except for Inner Mongolia) in China (31/32) has reported imported malaria cases in recent years [9, 35]. Without sufficient awareness and experience of handling imported malaria cases, most clinicians are facing new challenges in diagnosis and treatment of this type of malaria. Severe cases and even death have occurred in China every year. Surveys showed that in some areas, the proportion of patients diagnosed within 24 h of onset was only 13.3 % and the proportion of patients diagnosed at township-level hospitals was only 4.3 %. Misdiagnosis and missed diagnosis also occurred in some provincial/city hospitals [36], which undoubtedly brings the potential risk of reintroducing malaria into areas where transmission has been eliminated, but vectors still remain.

When planning for the next stage towards the elimination of malaria in China, the first point to emphasise is governmental support. Governments’ commitment at all levels should further strengthen and ensure substantial and consistent funding. The funding mechanisms of the Central Government need to be changed to address the new malaria situation. For example, funding coverage needs to be extended from historically malaria-endemic areas to 31 P/A/M, as imported cases are being reported in almost every part of China. In addition, the funding priority should shift to controlling malaria at border areas and the management of imported malaria. Secondly, new operational strategies need to be developed to achieve and maintain malaria elimination [37]. For example, network methods can be used to reach hard-to-reach populations such as migrant labourers and to improve the management of imported malaria [38]. New mechanisms for inter-sectorial cooperation and information exchange need to be further explored in order to deal with imported malaria more efficiently, such as through international cooperation at cross-border areas in Yunnan Province and neighbouring countries. This should include such factors as strengthening the village-level capacity for malaria diagnosis and treatment and county-level surveillance and response ability, and discovering malaria cases and managing them among the cross-border mobile population, thus reducing malaria on the Myanmar side. Other international collaborations such as through the Asia Pacific Malaria Elimination Network [39], the Asia Pacific Leaders Malaria Alliance [40], Chinese aid programmes for African countries and China’s Belt and Road Initiative will also be critical for the control of imported malaria; such collaborations will also contribute to malaria elimination in other countries by introducing China’s experiences, lessons and expertise in malaria control [41, 42]. In addition, operational researches need to be carried out for malaria elimination such as diagnostic methods for cases of malaria with low parasitemia, molecular techniques to distinguish local and imported cases, effective methods for monitoring drug resistance and susceptibility of local malaria vectors to imported cases [43]. Thirdly, an effective and sustainable malaria surveillance and response system tailored to local settings needs to be further developed [44]. Although no local malaria cases have been reported in most parts of China by 2014, malaria vectors still exist and the capacity of malaria control is still limited in some regions. This means that constant vigilance for imported cases will be important for the regions that are in the phase of preventing the reintroduction of malaria, especially in the cross-border region where importation risk is relatively high [45]. Fourthly, assessments for transmission risks need to be urgently conducted in order to develop targeted strategies and measures for surveillance and response in the regions that have passed the sub-national elimination assessment [46].

## Conclusion

The NMEP in China is successfully being implemented and the malaria map has been shrunk dramatically. The next five years are critical for achieving the goal of a completely malaria-free China by 2020. The priorities are malaria elimination at border areas, management of imported malaria, preventing malaria reintroduction, capacity building, and sustainability of malaria surveillance and response.

## Additional file

Additional file 1: Multilingual abstract in the six official working languages of the United Nations. (PDF 210 kb)

## Abbreviations

APCME: action plan of China malaria elimination
CISDCP: China information system for disease control and prevention
iRS: indoor residual spraying
LLIN: long-lasting insecticide-treated net
NMEP: National Malaria Elimination Programme
P/A/M: provinces autonomous regions and municipalities
