# Supplementary material for: Shrinking the malaria map in China: measuring the progress of the National Malaria Elimination Programme
Source: Infect Dis Poverty. 2016 May 19;5:52. doi: 10.1186/s40249-016-0146-5 (PMC4873993; doi:10.1186/s40249-016-0146-5)
Supplement: Additional file 1: — Multilingual abstract in the six official working languages of the United Nations. (PDF 210 kb) [file 40249_2016_146_MOESM1_ESM.pdf]

Translation of the abstract into the six official working languages of the United Nations

انخفض انتشار وباء الملا ريا في الصين: قياس التقدم المحرّز للبرنامج الوطني للقضاء على الملا ريا

تاو هو، ياو-باو ليو، شاو-سين تشانغ، تشي-اجي شيا، شوي-سين تشو، جون يان، جون تسو، تشان-تشون فنغ

ملخّص

تقديم عام: تم إحراز تقدم ملحوظ للقضاء على وباء الملاريا في الصين منذ انطلاق البرنامج الوطني للقضاء على الملاريا (NMEP) سنة 2010. قد سُجّل انخفاض هّام في معدل الإصابات بالملاريا محليّا إضافة إلى انخفاض عدد المناطق التي تشهد انتشارا لهذا المرض بشكل كبير. في المجموع، تّم الإبلاغ عن 3,078 حالة إصابة بالملاريا في عام 2014 ولكن لم يكن هناك سوى 56 حالة من السكان المحليين. وبهدف مواصلة تعزيز برنامج القضاء على هذا المرض فقد قمنا بمعاينة التقدّم المحرز والتجارب المرتبطة بالقضاء على هذا الوباء في الصين كما قمنا بتحديد التحديات والأولويات للمرحلة المقبلة من البرنامج.

التقنيات المستخدمة: تم جمع البيانات من شبكة نظام المعلومات الصيني لمراقبة الأمراض والوقاية منها ومن التقرير السنوي المتعلق بالقضاء على الملاريا وقد تم قياس التقدم المحرز للقضاء على هذا المرض من سنة 2010 إلى 2014.

النتائج: إثر تطبيق البرنامج الوطني للقضاء على الملاريا من سنة 2010 إلى 2014، شهد معدل الإصابة بهذا المرض انخفاضا بشكل مستمر وبقي انتشار هذا المرض على حاله في مقاطعة اليونان (الموجودة في الصين) وبمنطقة التبت المستقلة سنة 2014. وبحلول نهاية سنة 2015 أجرت 75.6% (1,636/2,163) من المقاطعات التي ينتشر فيها وباء الملاريا، التقييم دون الوطني للقضاء على المرض. تكمن أهّم التحديات في تلك الحالات الموجودة على الحدود وتلك المُتأتية من بلدان أخرى. يعتبر كل من الدعم المستدام والاستثمار من الحكومة وإنشاء مراقبة فعالة ونظام الاستجابة إضافة إلى تقييم المخاطر المحتملة في صورة ظهور هذا المرض مرة ثانية، أولويات للمرحلة القادمة من هذا البرنامج.

الخاتمة: تم تطبيق البرنامج الوطني للقضاء على الملاريا بنجاح حتى الآن وقد تقلص انتشار هذا المرض بشكل كبير، ومن بين أولويات هذا البرنامج هي التدخلات القائمة على منع انتقال هذا المرض إلى المناطق الحدودية وإدارة حالات الملاريا المتأتية من البلدان الأخرى ومنع إعادة انتشار هذا المرض من جديد وكذلك بناء القدرات ومواصلة مراقبة هذا المرض والاستجابة له بصفة مستدامة.

Translated from English version into Arabic by Zeineb Trabelsi, through

TRANSLATORS WITHOUT BORDERS

压缩中国的疟疾版图：国家消除疟疾行动的进展

胡桃，刘耀宝，张少森，夏志贵，周水森，严俊，曹俊，冯占春

摘要

引言：中国自2010年启动消除疟疾行动计划以来，消除疟疾工作取得了重要进展。全国本地疟疾发病率迅速下降，疟疾流行区范围显著缩小，2014年全国共报告疟疾病例3 078例，其中本地感染病例仅56例。为进一步推进消除疟疾工作，本文回顾了中国消除疟疾行动计划进展和经验，并对下阶段消除疟疾面临的挑战和工作重点进行了讨论。

方法：从中国疾病预防控制信息系统和全国消除疟疾工作年报收集有关数据，分析评价中国2010-2014年消除疟疾工作进展。

结果：自从 2010 年启动消除疟疾行动后到 2014 年，全国本地疟疾发病率持续下降，到 2014 年底，仅有云南省和西藏自治区报告有本地感染病例。2015 年底，全国有 75.6%（1 636/2 163）的疟疾流行县通过了消除疟疾考核。当前消除疟疾面临的主要挑战是边境疟疾和境外输入性疟疾。今后消除疟疾的工作重点是加强政府的持续支持和资金投入，建立有效监测与反应体系，以及疟疾再传播的风险评估。

结论：中国消除疟疾行动计划进展顺利，显著压缩了中国的疟疾版图。当前消除疟疾工作的优先领域包括阻断边境地区疟疾传播、输入性疟疾管理、防止疟疾输入再传播、能力建设以及监测与反应体系的维持。

Translated from English version into Chinese by Liu Yao-Bao

Réduire la carte du paludisme en Chine : mesure des progrès du Programme national d'élimination du paludisme

Tao Hu, Yao-Bao Liu, Shao-Sen Zhang, Zhi-Gui Xia, Shui-Sen Zhou, Jun Yan, Jun Cao, Zhan-Chun Feng

Résumé

Contexte: Depuis le lancement du Programme national d'élimination du paludisme (PNEP) en 2010, la Chine a fait des progrès considérables vers l'éradication de la maladie. L'incidence du paludisme acquis localement a diminué rapidement et les zones d'endémie se sont spectaculairement réduites. Au total, 3 078 cas de paludisme ont été rapportés en 2014 mais 56 seulement étaient indigènes. A l'appui du programme d'élimination, nous dressons un bilan de ses progrès et des retours d'expérience et identifions les difficultés et les priorités des prochaines étapes.

Méthodes: Nous avons recueilli les données du Système d'information chinois pour la lutte contre les maladies (sur le Web) et du Rapport annuel sur l'élimination du paludisme pour mesurer les progrès vers l'élimination du paludisme entre 2010 et 2014.

Résultats: Avec le déploiement du PNEP de 2010 à 2014, l'incidence locale du paludisme n'a pas cessé de décroître, restant élevée uniquement dans la province du Yunnan et la Région autonome du Tibet en 2014. À la fin de 2015, 75,6 % (1 636 sur 2 163) des circonscriptions de paludisme endémique ont réussi l'évaluation de l'élimination à l'échelle sub-nationale. Les principales difficultés concernent les cas frontaliers de paludisme et les cas importés d'autres pays. L'aide et les investissements continus du gouvernement, la mise en place d'un système efficace de surveillance et de réponse et l'évaluation des risques de réintroduction du paludisme seront les priorités de l'étape suivante du programme.

Conclusions: Le PNEP chinois a donné de bons résultats jusqu'ici et permis une réduction spectaculaire de l'aire de distribution du paludisme. Les priorités de l'élimination du paludisme concernent les interventions visant à stopper la transmission dans les régions frontalières, la gestion des cas importés, la prévention de la réintroduction, la constitution de capacités et le maintien dans la durée du système de surveillance et de réponse antipaludique.

Translated from English version into French by Suzanne Assenat, through

TRANSLATORS
WITHOUT BORDERS

Уменьшение малярийных областей на карте Китая: развитие Национальной программы по борьбе с малярией

Tao Hu, Yao-Bao Liu, Shao-Sen Zhang, Zhi-Gui Xia, Shui-Sen Zhou, Jun Yan, Jun Cao, Zhan-Chun Feng

Отрывок

Предпосылки: Со времени запуска Национальной программы по борьбе с малярией в 2010 году в Китае был отмечен большой успех в сфере борьбы с малярией. Количество случаев малярии стало быстро уменьшаться, область распространения малярии также значительно сократилась. Всего в 2014 году было сообщено о 3,078 случаях малярии, но только 56 были эндемическими. Чтобы способствовать дальнейшему развитию противомалярийной программы, мы рассмотрели прогресс и опыт, связанный с борьбой с малярией в Китае, определили цели и приоритеты для следующей стадии этой программы.

Методы: Данные были взяты из информационного вебпортала Китая, «Системы контроля и предотвращения и заболеваний», а также Ежегодного отчета по борьбе с малярией в Китае. Изучен прогресс уменьшения случаев малярии за период с 2010 по 2014 годы.
Результаты: В ходе внедрения Национальной программы по борьбе с малярией, в период 2010-2014 гг частота случаев заболевания малярией постоянно снижается, в 2014 году малярия наблюдалась только в провинции Юньнань и в автономном регионе Тибет. К концу 2015 г. 75.6% (1,636/2,163) малярийных эндемических округов, прошли внутреннюю национальную аттестацию. Основные проблемы – это случаи малярии в приграничных зонах и импортирование малярии из других стран. Неизменная поддержка и инвестиции правительства, внедрение эффективной системы контроля и реагирования, оценка риска возвращения малярии – главные приоритеты следующей ступени программы борьбы с малярией.
Выводы: Национальная программа по борьбе с малярией в Китае была внедрена успешно, зона распространения малярии значительно сократилась. Приоритетными задачами в борьбе с малярией являются введение поблочной передачи в приграничных зонах, случаи импорта малярии, предотвращение повторного возникновения малярии, строительство необходимых помещений и надежная система контроля и реагирования.

Translated from English version into Russian by Anna Philippova, through

TRANSLATORS
WITHOUT BORDERS

Estamos achicando el mapa de la malaria en China: Medición del progreso del Programa Nacional de Eliminación de la Malaria

Tao Hu, Yao-Bao Liu, Shao-Sen Zhang, Zhi-Gui Xia, Shui-Sen Zhou, Jun Yan, Jun Cao, Zhan-Chun Feng

Resumen

Antecedentes: Desde que se lanzó el Programa Nacional de Eliminación de la Malaria (NMEP) en el año 2010, se ha hecho un progreso bastante significativo hacia la eliminación de la malaria en China. La incidencia de casos de malaria localmente adquirida ha declinado rápidamente y las áreas endémicas también se han achicado dramáticamente. En el año 2014 se reportó un total de 3 078 casos de malaria, pero solo 56 casos fueron autóctonos. Para promover el programa de eliminación en mayor medida, hemos revisado el progreso y las experiencias asociadas con la eliminación de la malaria en China e identificado los desafíos y prioridades para la siguiente etapa del programa.
Métodos: Se recolectó información en línea del Sistema de Información para el Control y Prevención de Enfermedades y del Informe anual sobre la eliminación de la malaria en China. Se midió el progreso hacia la eliminación de la malaria entre los años 2010 y 2014.
Resultados: Durante la implementación del NMEP entre los años 2010 y 2014, la incidencia local de la malaria declinó de manera continua, solo quedando en el 2014 en la Provincia de Yunnan y en la Región Autónoma del Tíbet. Para fines del año 2015, 75,6% (1 636/2 163) de los condados donde la malaria es endémica pasaron la evaluación de eliminación sub-nacional. Los principales desafíos son los casos de malaria fronteriza y de malaria importada de otros países. El apoyo sostenible y la inversión por parte del gobierno, el establecimiento de un sistema efectivo de vigilancia y respuesta, y la evaluación del riesgo de posible reintroducción de la malaria son prioritarios para la siguiente etapa del programa de eliminación.
Conclusiones: Hasta ahora el NMEP fue implementado con éxito en China y el mapa de la malaria se ha achicado de manera significativa. Las prioridades para la eliminación de la malaria son intervenciones para bloquear la transmisión en las zonas fronterizas,

el manejo de casos importados de malaria, la prevención de la reintroducción de la malaria, el desarrollo de aptitudes y la sustentabilidad de la vigilancia de la malaria y su respuesta.

Translated from English version into Spanish by Maria Alejandra Aguada, through

TRANSLATORS
WITHOUT BORDERS
